# The Impact of the Central Asia Stunting Initiative on Stunting Among Children Under Five Years Old in Gilgit Baltistan and Chitral, Pakistan

**DOI:** 10.3390/nu17203255

**Published:** 2025-10-16

**Authors:** Imtiaz Hussain, Imran A. Chauhadry, Muhammad Umer, Noor Nisa, Sanober Nadeem, Mushtaq Hassan, Asma A. Sattar, Muhammad Atif Habib, Shabina Ariff, Aminah Jahangir, Claudia Hudspeth, Sajid B. Soofi, Zulfiqar A. Bhutta

**Affiliations:** 1 Centre of Excellence in Women and Child Health, Aga Khan University, Karachi 74800, Pakistan; imtiaz.hussain@aku.edu (I.H.); imran.ahmed@aku.edu (I.A.C.); muhammad.umer@aku.edu (M.U.);; 2 Department of Paediatrics & Child Health, Aga Khan University, Karachi 74800, Pakistan; 3 Aga Khan Foundation, Islamabad 92000, Pakistan; 4 Aga Khan Foundation, 1211 Geneva, Switzerland; aminah.jahangir@akdn.org (A.J.); claudia.hudspeth@akdn.org (C.H.); 5 Institute of Global Health & Development, Aga Khan University, Karachi 74800, Pakistan

**Keywords:** stunting, malnutrition, child nutrition, community-based interventions, Pakistan, Gilgit-Baltistan, Chitral

## Abstract

**Background**: Stunting, a form of chronic malnutrition, is a global health concern, especially in South Asia. Stunting remains a significant public health issue in Pakistan, particularly in remote regions like Gilgit-Baltistan and Chitral, where geographic isolation and socioeconomic challenges exacerbate malnutrition. The Aga Khan Development Network is leading the implementation of a program, Central Asia Stunting Initiative (CASI), with an aim to reduce stunting through community-driven maternal and child health interventions in the targeted areas of Gilgit Baltistan and Chitral. This study aimed to evaluate the effectiveness of CASI in improving child nutritional outcomes in Gilgit-Baltistan and Chitral. **Methods**: In this study, a single-group pre–post evaluation design was employed using baseline and midline cross-sectional surveys among households with children aged 0–59 months in Gilgit-Baltistan and Chitral. Data on child anthropometry, household food security, maternal education, and child feeding practices were collected from over 500 households using stratified sampling. **Results**: Results showed improvement in child health indicators between baseline and midline. Between baseline and midline, stunting declined from 40.9% to 35.4% in GBC (*p* = 0.02), with severe stunting dropping significantly (17.8% to 10.9%, *p* < 0.001). Wasting and underweight rates also showed marked reductions. Improvements in breastfeeding rates (71.3% to 88.3%) and dietary diversity (4.0% to 26.8%) were observed. However, food security declined sharply from 95.2% to 11.9%, underscoring persistent economic stress. **Conclusions**: CASI interventions yielded substantial improvements in child nutrition and maternal behaviours. However, sustained progress requires integrated strategies addressing food insecurity, economic empowerment, and long-term resilience. Future programs should adopt a multi-sectoral approach to tackle chronic malnutrition comprehensively. Despite this, results indicated an overall improvement due to CASI interventions, signifying the importance of integrated, community-based approaches in addressing stunting.

## 1. Introduction

Stunting, a form of chronic malnutrition, poses a significant public health challenge worldwide, especially in developing countries [1,2]. It is marked by impaired linear growth in children under five due to prolonged nutritional deficiencies, repeated infections, and insufficient psychosocial stimulation [3]. The World Health Organization (WHO) estimates that approximately 22% of children under five globally suffer from stunting, with the burden disproportionately affecting low- and middle-income countries [1,4]. Stunting has immediate health implications and long-term consequences on cognitive development, economic productivity, and overall well-being, thereby perpetuating cycles of poverty and underdevelopment [5,6,7].

The impact of stunting is especially severe in South Asia, where malnutrition remains a significant challenge despite economic growth and development initiatives [1,4]. Pakistan, the fifth most populous country in the world, has one of the highest rates of childhood stunting, with nearly 40.2% of children under five affected, according to the National Nutrition Survey [8]. Among various regions in Pakistan, Gilgit-Baltistan and Chitral face unique geographical and socio-economic challenges that aggravate malnutrition and stunting rates [8,9]. The survey indicated that the prevalence of stunting in Gilgit-Baltistan is 46%, surpassing the national average. While specific data for Chitral is not separately reported in the study, it falls within Khyber Pakhtunkhwa (KP), where the stunting rate is 40% [8,9]. These figures highlight the potential for targeted nutritional interventions to address the high rates of stunting in these regions.

Stunting during childhood has far-reaching consequences that extend beyond physical growth. Research shows that children who experience stunting are at a greater risk of poor educational outcomes due to impaired cognitive development [10]. This, in turn, affects their economic potential in adulthood, leading to lower earnings and productivity, thereby contributing to the cycle of poverty [11]. Furthermore, stunting is linked to an increased susceptibility to non-communicable diseases (NCDs) in adulthood, including obesity, diabetes, and cardiovascular diseases [12]. According to Soofi et al. [9], male children and those from households lacking access to improved sanitation facilities are particularly vulnerable to stunting. Given these long-term effects, addressing stunting is not merely a matter of child health but is also crucial for sustainable development [7].

Tackling stunting in Gilgit-Baltistan and Chitral requires a multi-sectoral approach that integrates health, nutrition, education, and social protection programs. The Government of Pakistan, in collaboration with international organizations such as UNICEF and the World Food Program (WFP), has introduced initiatives like the Pakistan Multi-Sectoral Nutrition Strategy (2018–2025) to combat malnutrition [13,14]. Moreover, these regions have unique geographical challenges and socio-cultural practices that include but are not limited to reliance on low diversity diets and taboos surrounding women reproductive health. Therefore, these areas require localized, community-driven interventions.

Studies have demonstrated that nutrition interventions based on the 1000-day approach and supplementation strategies significantly improve nutrition indicators among women and children [15,16], promising a healthier future generation. It has also been recognized that adequate maternal nutrition through iron and folic acid supplementation lowers the risk of maternal anemia and low birth weight. At the same time, early initiation of breastfeeding and exclusive breastfeeding contribute to improved child survival and development [17]. Similarly, Vitamin A supplementation, ready-to-use therapeutic foods (RUTF), and micronutrient powders have been shown to reduce childhood wasting, stunting, morbidity, and mortality [17]. Community-based programs implementing this approach, such as those in South Asia and sub-Saharan Africa, have successfully lowered malnutrition rates and improved maternal and child health [16,18,19].

Acknowledging the pressing need to address malnutrition, Pakistan has rolled out targeted programs for mothers and children to improve nutrition. One such effort is the Central Asia Stunting Initiative (CASI), spearheaded by the Aga Khan Development Network and the Aga Khan Foundation (AKF) in partnership with essential government agencies [20]. The policy was formed as a two-pronged approach focusing on the 1000 days window from pregnancy to the second birthday of a child, with continual growth monitoring until five years of age and focusing on preconceptual nutrition of mothers. The CASI project aims to decrease stunting and enhance maternal and child health through community-driven interventions that focus primarily on women of reproductive age and children under five, particularly those under two years old (Table 1) [20]. This initiative is being implemented in Gilgit Baltistan and Chitral, which were chosen for their alarming stunting rates [8].

Although existing programs like Multi-Sectoral Nutrition Strategy (2018–2025) have been implemented in the country, studies show limited evidence on the effectiveness of community-based interventions in the remote mountainous areas. This study presents an independent external evaluation of the nutritional interventions under CASI on reducing stunting rates among children under five years of age in Gilgit-Baltistan and Chitral.

## 2. Methodology

The Aga Khan Development Network (AKDN) implemented the intervention package detailed in Table 1. CASI implementation in GBC took place between 2019 and 2023; the baseline survey was conducted from October to December 2019, followed by midline survey from September to November 2023 to capture the impact of the program.

An independent group of researchers from Aga Khan University in Pakistan evaluated these interventions. Given that CASI interventions were applied to a broad population in the Gilgit Baltistan province and Chitral district, and due to the nature of the intervention, no demographic segment could be excluded from receiving benefits; thus, our study does not include a control group. To address this issue, we adopted a Single-Group Pre–Post Test Design [21] for assessing the effectiveness of the CASI intervention package. In our study, participants served as their own controls, comparing baseline (pre-intervention) data with mid-intervention data to evaluate shifts in nutritional indicators.

Our baseline and midline survey evaluation focused on children aged 0–59 months, with stunting as the primary outcome. A varying sample size is present across different indicators because of the age-specific eligibility and consent withdrawal for anthropometry. This deviation is acknowledged as a study limitation. Given a 45% prevalence of stunting among children under five, we estimated a sample size of 500 households with at least one child under five to meet the primary objective of reducing stunting. A 95% confidence level and a 5% margin of error were utilized, including a 20% non-response rate, to achieve this sample size [22]. We used a two-stage stratified sampling design, treating villages as primary sampling units (PSUs) and households within those villages as secondary sampling units (SSUs). Villages were selected using probability proportional to size sampling (PPS), while households were identified through systematic random sampling. A new household listing was conducted to ensure an accurate selection, prioritizing those with children under the age of five. Respondent selection followed specific criteria. Household heads or knowledgeable individuals aged 18 or older contributed data on demographics, socioeconomic conditions, and food security. Household food security was assessed using the Household Food Insecurity Access Scale (HFIAS), which categorizes households into food secure, mildly insecure, moderately insecure, and severely insecure. Women in selected households were surveyed for reproductive health and dietary information. Data for children aged 0–59 months were gathered from mothers or caretakers, including anthropometric measurements for a randomly chosen child (6–59 months) per household during the baseline survey and all available children under five during the midline survey. Height-for-age z-scores (HAZ) were calculated for children over two years, while length-for-age z-scores (LAZ) were derived for those under two. According to WHO classifications, children were designated as stunted, wasted, and underweight, ensuring alignment with global standards [23].

The research and evaluation team at Aga Khan University created and reviewed an extensive survey protocol. Data collection tools were designed, translated, and pre-tested for accuracy. The survey was conducted by independent teams from Aga Khan University, trained in data collection, anthropometry, and Hb testing. Survey quality was monitored at various levels, with team leaders supervising data collection, anthropometry, and Hb testing in the field. To ensure accuracy, anthropometric tools were calibrated daily before field visits and standardized using intra- and inter-observer reliability tests. AKU’s Data Management Unit (DMU) provided real-time dashboard tracking and data validation. Regular feedback was given to supervisors to address inconsistencies and improve data quality. Data collection employed Computer-Assisted Personal Interviews (CAPI) using handheld tablets. At the same time, paper-based personal interviews (PAPI) were conducted in security-sensitive areas, with the subsequent data being transcribed into the system. Data entry utilized a custom Android application featuring built-in validation checks, and a web-based dashboard facilitated real-time oversight. Data security was upheld through encryption and limited access. The analysis was performed using STATA, considering stunting (height/length-for-age z-score of <−2) as an outcome variable for children under five. Statistical tests, including chi-square tests for categorical variables and *t*-tests for continuous variables, assessed pre- and post-intervention differences, determining significance with *p*-values (*p* < 0.05). Cohen’s h [24] was utilized to measure effect size, quantifying the magnitude of change between proportions.

The Ethical Review Committee at Aga Khan University approved the study (ERC# 2019-1582-4219 approval date 3 July 2019). The research team ensured confidentiality by obtaining informed consent from all participants. They referred children with severe malnutrition or illnesses to health facilities for necessary treatment.

## 3. Results

The study provides a comparative analysis of baseline and midline data, highlighting the progress and challenges in household, maternal, and child health indicators. The size of households remained stable during both periods. However, socioeconomic disparities grew, evident in a slight increase in the proportion of the poorest families (from 20.1% to 21.5%) and a decrease in the wealthiest households (from 19.9% to 18.7%). Housing conditions showed mixed results; while the percentage of homes with finished walls rose (from 50.5% to 65.4%), finished roofing experienced a significant decline (from 41.9% to 11.1%), accompanied by a sharp rise in rudimentary roofing (from 28.5% to 88.8%). Access to safe drinking water decreased slightly (99.1% to 98.2%), whereas improved sanitation facilities increased from 96.8% to 99.0%. The most concerning change was the drastic drop in food security, with the proportion of food-secure households plummeting from 95.2% to 11.9%. Meanwhile, moderate to severe food insecurity rose from 3.5% to 51.6%, indicating a significant decline in food availability and access.

Maternal indicators showed notable educational improvements, as maternal literacy rose from 50.0% to 67.9%, and paternal literacy increased from 64.2% to 79.7%. However, maternal employment remained low, with only a slight rise from 4.9% to 5.2%. Child health indicators demonstrated positive trends, especially in breastfeeding rates, which increased from 71.3% to 88.3%, and dietary diversity among children aged 6–24 months, improving from 4.0% to 26.8%. Immunization coverage remained consistently high, with fully immunized children representing 99.1% at midline, although a small proportion of partially (0.6%) and non-immunized (0.4%) children appeared in the midline data. Despite improvements in education, sanitation, and child nutrition, the decline in food security and worsening housing conditions underscore significant economic challenges, emphasizing the urgent need for policy interventions (Table 2).

The comparative analysis of baseline and midline data on child nutritional status in Gilgit-Baltistan & Chitral (GBC), revealed notable improvements in nutrition indicators, especially regarding underweight, stunting, and wasting. In GBC, the rate of underweight children decreased from 20.8% to 15.5% (*p* = 0.004, effect size = 0.14), while severe underweight cases dropped from 7.0% to 2.9% (*p* < 0.001, effect size = 0.19) showing a significant reduction in both. Stunting rates fell from 40.9% to 35.4% (*p* = 0.02, effect size = 0.11), and severe stunting significantly reduced from 17.8% to 10.9% (*p* < 0.001, effect size = 0.20). Similarly, the prevalence of wasting decreased from 9.8% to 4.9% (*p* < 0.001, effect size = 0.19), with severe wasting dropping from 5.0% to 1.5% (*p* < 0.001, effect size = 0.21), indicating statistically significant results. However, concurrent stunting and wasting rates changed insignificantly (from 1.4% to 1.6%, *p* = 0.752, effect size = 0.02); see Table 3. These patterns indicate a noteworthy progress in combating both acute and chronic malnutrition in GBC, likely due to enhanced healthcare, dietary programs, and food security measures.

In Chitral, similar positive trends were noted, with underweight prevalence decreasing from 21.5% to 17.9%, even though this change was not statistically significant (*p* = 0.125, effect size = 0.09). Severe underweight rates, however, declined significantly from 8.4% to 3.7% (*p* = 0.001, effect size = 0.20). Stunting prevalence dropped from 46.0% to 38.8% (*p* = 0.016, effect size = 0.15), while severe stunting saw a considerable decline from 22.3% to 13.1% (*p* < 0.001, effect size = 0.24). Wasting prevalence was reduced from 10.9% to 5.1% (*p* = 0.001, effect size = 0.22), and severe wasting decreased from 5.0% to 1.3% (*p* = 0.001, effect size = 0.22), giving statistically significant results. Nonetheless, concurrent stunting and wasting rates showed no significant shift (from 1.7% to 2.0%, *p* = 0.694, effect size = 0.02). These results indicate notable reductions in severe forms of malnutrition in Chitral, likely due to targeted interventions, improved maternal health, and better infant feeding practices (Table 3).

In GB, underweight prevalence fell from 19.8% to 11.9% (*p* = 0.007, effect size = 0.22), and severe underweight cases dropped from 4.9% to 1.7% (*p* = 0.026, effect size = 0.18). The stunting prevalence reflected only a minor decrease from 33.1% to 30.3% (*p* = 0.456, effect size = 0.06), and severe stunting declined from 10.9% to 7.6% (*p* = 0.166, effect size = 0.11) showing both values to be statistically insignificant. Wasting prevalence fell from 8.2% to 4.6% (*p* = 0.08, effect size = 0.15), and severe wasting significantly reduced from 4.9% to 1.7% (*p* = 0.031, effect size = 0.18). Conversely, concurrent stunting and wasting rates remained stable at 1.0% (*p* = 0.98, effect size = 0.00); see Table 3. While GB showed some improvement in acute malnutrition, the ongoing levels of stunting highlight a continued problem with chronic malnutrition, underscoring the necessity for enduring nutrition programs and long-term strategies.

## 4. Discussion

The findings of this study demonstrate that the CASI has significantly reduced rates of stunting, underweight, and wasting among children under five in Gilgit-Baltistan and Chitral. This progress in nutrition indicators highlights the effectiveness of targeted nutritional initiatives, maternal health education, and improved child-feeding practices. However, persistent high stunting rates, declining food security, and deteriorating housing conditions reveal ongoing socioeconomic challenges that continue to affect child nutrition outcomes.

These results align with trends observed in similar nutritional interventions across South Asia. In Bangladesh, programs prioritizing maternal nutrition, dietary diversity, and community healthcare have successfully reduced stunting despite ongoing food security challenges [25]. In India, the Integrated Child Development Services (ICDS) program has improved underweight and wasting rates, but poverty and sanitation concerns have hindered progress in reducing stunting [26]. Meanwhile, Nepal’s Multi-Sector Nutrition Plan (MSNP) has achieved more substantial success by integrating food security initiatives, sanitation improvements, and social protection measures [27]. CASI stands out for its community-driven approach and rigorous monitoring, which sets it apart from ongoing programs in Pakistan. Moreover, the integration of maternal child health with WASH interventions is also a distinctive feature of the program.

Our analysis of the findings confirms that CASI has successfully reduced severe stunting and wasting by enhancing access to healthcare, promoting maternal education, and encouraging healthier dietary behaviours. Despite these improvements, the prevalence of stunting in Gilgit-Baltistan (30.3%) remains higher than in Nepal (25.8%) and certain parts of India (27.5%), highlighting the persistence of structural barriers such as geographic isolation and economic disparities [26,27]. Although CASI has improved nutritional outcomes, the decline in food security (from 95.2% to 11.9%) raises concerns about the program’s long-term sustainability. However, this may partly be explained by the overlap of the program with the period of COVID-19 and subsequent floods. These natural disasters led to hyperinflation, job losses, reverse migration, and increased poverty. Moreover, the destruction of crops and livestock resulted in increased dependence on market purchases and higher food prices, thus exacerbating food insecurity.

The potential causal interactions between CASI interventions and observed improvements can be explored through various pathways. Improved maternal nutrition and dietary diversity lead to better birth outcomes, resulting in reduced neonatal morbidity and improved child growth [17]. Increased access to fortified foods and dietary supplementation directly improves nutrient intake, decreasing malnutrition and micronutrient deficiency and supporting cognitive and physical development [28]. Additionally, improved sanitation and hygiene reduce exposure to infections, which can exacerbate malnutrition [29].

The findings of this study highlight the importance of community-driven interventions and awareness programs that enhance knowledge on appropriate infant and young child feeding practices and strengthen health systems by improving maternal literacy and engaging local structures. These interventions can further be optimized by ensuring the availability of reliable anthropometric tools and well-trained community health workers to reduce attrition and maintain program quality [30]. Moreover, the program highlights the need to complement health-focused interventions with strategies that address food security and economic empowerment, along with agricultural policies for lasting impact.

Our study employs a single-group pre–post test design, allowing for a direct comparison between baseline and midline data. The substantial sample size and stratified sampling method enhance the reliability of the findings, while Computer-Assisted Personal Interviews (CAPI) improve data accuracy. However, the absence of a control group prevents definitive causal inferences, and the study’s short duration limits insights into long-term effects. Additionally, the analysis does not fully account for regional differences in dietary practices and healthcare access, which may affect the broader applicability of the conclusions. We also acknowledge that this study design is subject to potential biases that include seasonal variations and migration that may lead to varied intervention effectiveness.

To sustain progress, future efforts must integrate economic development with nutritional initiatives supported by government and encouraging cross-sector collaboration. Policymakers should strengthen food security by adequate resource allocation, introducing subsidies, expanding agricultural programs, and improving market access. At the same time, enhancing sanitation and hygiene education can help combat malnutrition related to infections. Furthermore, gender inequalities observed in livelihood suggest that nutrition programs may benefit from women empowerment initiatives. Strengthening social safety nets and promoting community-led livelihood programs could also build resilience against economic hardship and food shortages. Long-term monitoring and adopting multi-sectoral strategies, such as Nepal’s MSNP, could further enhance CASI’s effectiveness. Moreover, CASI’s distinctive community-driven approach with real-time monitoring offers the potential for replication in other resource-constrained regions. Future studies could include longitudinal tracking to assess nutritional gains over time to ensure sustenance without external support.

## 5. Conclusions

In conclusion, CASI has made a significant improvement in child nutrition in Gilgit-Baltistan and Chitral. The reductions in severe malnutrition demonstrate the success of maternal education, child-feeding practices, and healthcare advancement. However, persistent food security and economic challenges indicate the need for more integrated policies. A comparative review with neighbouring countries suggests that incorporating food security measures and long-term financial support could amplify CASI’s impact. Future strategies should prioritize holistic, multi-sectoral approaches to ensure sustained improvements in child nutrition without external support.

## Figures and Tables

**Table 1:** Interventions Implemented Under the Central Asia Stunting Initiative (CASI).

Target Group	Key Interventions
Pregnancy and Lactating Women	- Antenatal and postnatal care and nutritional assessment - Provision of energy-dense nutritious foods (LNS) - Iron and folic acid supplementation - Intensive nutrition counseling and behavioral change communication - Follow-ups
Birth to 6 months	- Helping Baby Breathe - Helping Baby Survive - Promotion of exclusive breastfeeding - Immunization - Behavioral change communication (IYCF practices, care practices, hygiene, and sanitation) - Follow-ups
Children (6–23 months)	- Promotion of continued breastfeeding - Immunization - Promotion of Infant and Young Child Feeding (IYCF) practices - Provision of LNS and iron supplementation - Growth monitoring - Vitamin A supplementation - Treatment of acute malnutrition (SAM and MAM) - Behavioral change communication (healthy diets, food demonstrations, care practices, hygiene, and sanitation)
Children (24–59 months)	- Growth monitoring - Improved hygiene and sanitation practices - Treatment of acute malnutrition (SAM and MAM) with LNS and RUTF - Follow-ups - Behavioral change communication (healthy diets, care practices, hygiene, and sanitation)

**Table 2:** Sociodemographic and Health Indicators, Baseline and Midline Surveys.

Variable	Baseline (% (95% CIs))	Midline (% (95% CIs))
Household-level indicators	N = 1022	N = 961
Household Size; mean (95% CIs)	7.4 (7.2–7.6)	7.5 (7.3–7.7)
SES Status		
Poorest	20.1% (17.6–22.6%)	21.5% (18.6–24.4%)
Poor	19.9% (17.4–22.4%)	20.5% (17.7–23.4%)
Middle	20.0% (17.5–22.6%)	19.2% (16.5–21.9%)
Rich	20.0% (17.4–22.6%)	20.1% (17.3–22.9%)
Richest	19.9% (17.4–22.5%)	18.7% (16.1–21.3%)
Type of Housing		
Flooring		
Natural	35.2% (32.2–38.2%)	37.7% (34.3–41.1%)
Rudiment	1.9% (1.0–2.8%)	0.6% (0.1–1.0%)
Finished	61.0% (57.9–64.0%)	61.7% (58.3–65.1%)
Other	1.9% (1.0–2.8%)	-
Roof		
Natural roofing	0.1% (0.0–0.4%)	0.2% (0.0–0.4%)
Rudimentary roofing	28.5% (25.6–31.5%)	88.8% (86.7–90.8%)
Finished roofing	41.9% (38.7–45.0%)	11.1% (9.0–13.1%)
Other	29.4% (26.6–32.3%)	-
Walls		
Natural walls	7.4% (5.8–9.0%)	0.8% (0.2–1.4%)
Rudimentary walls	41.1% (37.9–44.2%)	33.8% (30.5–37.1%)
Finished walls	50.5% (47.3–53.7%)	65.4% (62.1–68.7%)
Other	1.0% (0.3–1.7%)	-
Access to Safe Drinking Water (Improved sources of water)	99.1% (98.4–99.8%)	98.2% (97.3–99.1%)
Improved Sanitation Facilities	96.8% (95.7–98.0%)	99.0% (98.2–99.8%)
Food Security Status	N = 1022	N = 961
Food Secure	95.2% (93.7–96.7%)	11.9% (9.7–14.0%)
Mild Food Insecure	1.3% (0.5–2.2%)	36.6% (33.2–39.9%)
Moderate Food Insecure	1.2% (0.4–1.9%)	44.1% (40.6–47.5%)
Severely Food Insecure	2.3% (1.3–3.4%)	7.5% (5.7–9.3%)
Mothers level indicators	N = 827	N = 1259
Mother’s Age Group		
Less than 20 years	2.3% (1.2–3.4%)	2.7% (1.7–3.8%)
20–34 years	77.1% (74.1–80.2%)	73.7% (71.0–76.4%)
35–49 years	20.6% (17.6–23.5%)	23.6% (21.0–26.2%)
Mother’s Education Level		
Illiterate	50.0% (46.4–53.6%)	32.1% (29.2–34.9%)
Literate	50.0% (46.4–53.6%)	67.9% (65.1–70.8%)
Employment Status of Mother		
Working	4.9% (3.4–6.5%)	5.2% (3.9–6.5%)
Not working	95.1% (93.5–96.6%)	94.8% (93.5–96.1%)
Father’s Education Level	N = 785	N = 1261
Illiterate	35.8% (32.3–39.4%)	20.3% (17.8–22.8%)
Literate	64.2% (60.6–67.7%)	79.7% (77.2–82.2%)
Child level indicators	N = 843	N = 1286
Child’s Sex		
Male	47.7% (44.2–51.3%)	48.9% (45.9–51.9%)
Female	52.3% (48.7–55.8%)	51.1% (48.1–54.1%)
Child’s Age Group		
0–5 months	9.9% (7.8–12.1%)	8.9% (7.1–10.6%)
6–11 months	9.2% (7.1–11.2%)	9.9% (8.0–11.7%)
12–23 months	15.1% (12.6–17.7%)	18.1% (15.8–20.4%)
24–35 months	25.6% (22.5–28.6%)	23.4% (20.8–25.9%)
36–47 months	21.3% (18.4–24.3%)	19.7% (17.3–22.1%)
48–59 months	18.8% (16.1–21.6%)	20.1% (17.7–22.5%)
Child’s Breastfeeding Status	N = 80	N = 108
	71.3% (61.3–81.3%)	88.3% (81.7–95.0%)
MDD (6–24 month) IYCF	N = 207	N = 360
	4.0% (1.2–6.8%)	26.8% (21.9–31.8%)
Immunization Status of Child (12–23 months of age)	N = 126	N = 235
Not immunized	-	0.4% (0.0–0.9%)
Partially immunized	-	0.6% (0.0–1.4%)
Fully immunized	100.0% (100.0–100.0%)	99.1% (98.1–100.0%)

**Table 3:** Difference in Nutrition Status among children, *p* Values, and Effect Size Baseline vs. Midline Surveys.

	Baseline-% (95% CIs)	N	Midline-% (95% CIs)	N	Midline-Baseline (% Diff) (95% CIs)	*p* Value	Effect Size Cohens h
Nutritional status							
GBC							
Underweight	20.8% (18.0–23.7%)	842	15.5% (13.3–17.7%)	1275	−5.4% (−9.0–−1.8%)	0.004	0.14
Severely Underweight	7.0% (5.3–8.8%)	842	2.9% (1.9–3.9%)	1275	−4.2% (−6.2–−2.1%)	<0.001	0.19
Stunted	40.9% (37.2–44.5%)	767	35.4% (32.5–38.2%)	1271	−5.5% (−10.2–−0.9%)	0.02	0.11
Severely Stunted	17.8% (15.0–20.5%)	767	10.9% (9.0–12.7%)	1271	−6.9% (−10.3–−3.6%)	<0.001	0.20
Wasted	9.8% (7.6–12.0%)	778	4.9% (3.5–6.2%)	1270	−5.0% (−7.5–−2.4%)	<0.001	0.19
Severely Wasted	5.0% (3.4–6.6%)	778	1.5% (0.7–2.2%)	1270	−3.5% (−5.3–−1.7%)	<0.001	0.21
Concurrent Stunting & Wasting	1.4% (0.6–2.2%)	734	1.6% (0.8–2.4%)	1266	0.2% (−0.9–1.3%)	0.752	0.02
Chitral							
Underweight	21.5% (18.0–24.9%)	570	17.9% (14.9–20.9%)	745	−3.6% (−8.2–1.0%)	0.125	0.09
Severely Underweight	8.4% (6.1–10.7%)	570	3.7% (2.2–5.2%)	745	−4.7% (−7.4–−1.9%)	0.001	0.20
Stunted	46.0% (41.6–50.5%)	512	38.8% (35.0–42.6%)	743	−7.2% (−13.1–−1.4%)	0.016	0.15
Severely Stunted	22.3% (18.6–26.0%)	512	13.1% (10.4–15.7%)	743	−9.3% (−13.8–−4.7%)	<0.001	0.24
Wasted	10.9% (8.1–13.7%)	527	5.1% (3.3–6.9%)	743	−5.8% (−9.1–−2.5%)	0.001	0.22
Severely Wasted	5.0% (3.0–7.0%)	527	1.3% (0.4–2.3%)	743	−3.6% (−5.9–−1.4%)	0.001	0.22
Concurrent Stunting & Wasting	1.7% (0.6–2.9%)	492	2.0% (0.9–3.2%)	740	0.3% (−1.3–1.9%)	0.694	0.02
GB							
Underweight	19.8% (14.8–24.7%)	272	11.9% (8.8–14.9%)	530	−7.9% (−13.7–−2.1%)	0.007	0.22
Severely Underweight	4.9% (2.3–7.5%)	272	1.7% (0.6–2.7%)	530	−3.2% (−6.1–−0.4%)	0.026	0.18
Stunted	33.1% (27.0–39.2%)	255	30.3% (25.9–34.6%)	528	−2.8% (−10.3–4.6%)	0.456	0.06
Severely Stunted	10.9% (6.9–14.9%)	255	7.6% (5.1–10.1%)	528	−3.3% (−8.0–1.4%)	0.166	0.11
Wasted	8.2% (4.7–11.7%)	251	4.6% (2.5–6.6%)	527	−3.6% (−7.7–0.4%)	0.08	0.15
Severely Wasted	4.9% (2.2–7.7%)	251	1.7% (0.5–2.9%)	527	−3.2% (−6.2–−0.3%)	0.031	0.18
Concurrent Stunting & Wasting	1.0% (−0.1–2.1%)	242	1.0% (0.1–1.8%)	526	0.0% (−1.4–1.4%)	0.98	0.00

## Data Availability

The raw data supporting the conclusions of this article will be made available by the authors on request.

## References

[1] World Health Organization Malnutrition.

[2] Kramer C.V., Allen S. (2015). Malnutrition in developing countries. Paediatr. Child Health.

[3] De Onis M., Branca F. (2016). Childhood stunting: A global perspective. Matern. Child Nutr..

[4] UNICEF, World Health Organization (2020). Levels and Trends in Child Malnutrition: Key Findings of the 2019 Edition of the Joint Child Malnutrition Estimates.

[5] Black R.E., Victora C.G., Walker S.P., Bhutta Z.A., Christian P., de Onis M., Ezzati M., Grantham-McGregor S., Katz J., Martorell R. (2013). Maternal and child undernutrition and overweight in low-income and middle-income countries. Lancet.

[6] Soliman A., De Sanctis V., Alaaraj N., Ahmed S., Alyafei F., Hamed N., Soliman N. (2021). Early and long-term consequences of nutritional stunting: From childhood to adulthood. Acta Biomed..

[7] Leroy J.L., Frongillo E.A. (2019). Perspective: What does stunting really mean? A critical review of the evidence. Adv. Nutr..

[8] Government of Pakistan, UNICEF (2019). National Nutrition Survey 2018—Key Findings Report.

[9] Soofi S.B., Khan A., Kureishy S., Hussain I., Habib M.A., Umer M., Ariff S., Muhammad S., Rizvi A., Ahmed I. (2023). Determinants of Stunting Among Children Under Five in Pakistan. Preprints.

[10] Grantham-McGregor S., Cheung Y.B., Cueto S., Glewwe P., Richter L., Strupp B. (2007). Developmental potential in the first 5 years for children in developing countries. Lancet.

[11] Hoddinott J., Alderman H., Behrman J.R., Haddad L., Horton S. (2013). The economic rationale for investing in stunting reduction. Matern. Child Nutr..

[12] Victora C.G., Adair L., Fall C., Hallal P.C., Martorell R., Richter L., Sachdev H.S. (2008). Maternal and child undernutrition: Consequences for adult health and human capital. Lancet.

[13] Government of Pakistan (2018). Pakistan Multi-Sectoral Nutrition Strategy 2018–2025.

[14] Ahmed T., Hossain M., Mahfuz M., Choudhury N., Ahmed S. (2019). Imperatives for reducing child stunting in Bangladesh. Matern Child Nutr..

[15] English R., Peer N., Honikman S., Tugendhaft A., Hofman K.J. (2017). ‘First 1000 days’ health interventions in low- and middle-income countries: Alignment of South African policies with high-quality evidence. Glob. Health Action.

[16] Soofi S.B., Khan G.N., Ariff S., Ihtesham Y., Tanimoune M., Rizvi A., Sajid M., Garzon C., de Pee S., Bhutta Z.A. (2022). Effectiveness of nutritional supplementation during the first 1000 days of life to reduce child undernutrition: A cluster randomized controlled trial in Pakistan. Lancet Reg. Health Southeast Asia.

[17] Bhutta Z.A., Das J.K., Rizvi A., Gaffey M.F., Walker N., Horton S., Webb P., Lartey A., Black R.E. (2013). Evidence-based interventions for improvement of maternal and child nutrition: What can be done and at what cost?. Lancet.

[18] Taneja S., Chowdhury R., Dhabhai N., Upadhyay R.P., Mazumder S., Sharma S., Bhatia K., Chellani H., Dewan R., Mittal P. (2022). Impact of a package of health, nutrition, psychosocial support, and WaSH interventions delivered during preconception, pregnancy, and early childhood periods on birth outcomes and linear growth at 24 months: Factorial, individually randomized controlled trial. BMJ.

[19] Kulwa K.B., Verstraeten R., Bouckaert K.P., Mamiro P.S., Kolsteren P.W., Lachat C. (2014). Effectiveness of a nutrition education package in improving feeding practices, dietary adequacy and growth of infants and young children in rural Tanzania: Rationale, design and methods of a cluster randomized trial. BMC Public Health.

[20] Aga Khan Development Network (2022). Central Asia Stunting Initiative (CASI) Annual Report 2022. https://akdnehrc.com/annual-reports/2022/casi.html.

[21] Centre for Epidemiology and Evidence (2023). Study Design for Evaluating Population Health and Health Service Interventions: A Guide.

[22] Cochran W.G. (1963). Sampling Techniques.

[23] World Health Organization (2009). AnthroPlus for Personal Computers Manual: Software for Assessing Growth of the World’s Children and Adolescents.

[24] Sullivan G.M., Feinn R. (2012). Using effect size—Or why the P value is not enough. J. Grad. Med. Educ..

[25] Islam M.R., Rahman M.S., Rahman M.M., Nomura S., De Silva A., Lanerolle P., Jung J., Rahman M.M. (2020). Reducing childhood malnutrition in Bangladesh: The importance of addressing socioeconomic inequalities. Public Health Nutr..

[26] Kapil U. (2002). Integrated Child Development Services (ICDS) scheme: A program for holistic development of children in India. Indian J. Pediatr..

[27] Ruducha J., Bhatia A., Mann C., Torlesse H. (2022). Multisectoral nutrition planning in Nepal: Evidence from an organizational network analysis. Matern. Child Nutr..

[28] Tam E., Keats E.C., Rind F., Das J.K., Bhutta Z.A. (2020). Micronutrient supplementation and fortification interventions on health and development outcomes among children under five in low- and middle-income countries: A systematic review and meta-analysis. Nutrients.

[29] Perdomo C.D., Rodríguez E.R., Magallanes H.C., Flores Navarro H.E., Matul Pérez S.E., Moyano D. (2019). Impact of a community program for child malnutrition. Rev. Chil. Pediatr..

[30] Wells J.C., Sawaya A.L., Wibaek R., Mwangome M., Poullas M.S., Yajnik C.S., Demaio A. (2020). The double burden of malnutrition: Aetiological pathways and consequences for health. Lancet.

